# Toward a Common Secure Future: Four Global Commissions in the Wake of Ebola

**DOI:** 10.1371/journal.pmed.1002042

**Published:** 2016-05-19

**Authors:** Lawrence O. Gostin, Oyewale Tomori, Suwit Wibulpolprasert, Ashish K. Jha, Julio Frenk, Suerie Moon, Joy Phumaphi, Peter Piot, Barbara Stocking, Victor J. Dzau, Gabriel M. Leung

**Affiliations:** 1 O’Neill Institute for National and Global Health Law, Georgetown University, Washington, DC, United States of America; 2 Nigeria Academy of Sciences, Lagos, Nigeria; 3 Ministry of Public Health, Nonthaburi, Thailand; 4 Harvard T.H. Chan School of Public Health, Boston, Massachusetts, United States of America; 5 University of Miami, Coral Gables, Florida, United States of America; 6 Harvard Global Health Institute, Harvard University, Cambridge, Massachusetts, United States of America; 7 African Leaders Malaria Alliance, New York, New York, United States of America; 8 London School of Hygiene & Tropical Medicine, London, United Kingdom; 9 Murray Edwards College, University of Cambridge, United Kingdom; 10 National Academy of Medicine, Washington, DC, United States of America; 11 Li Ka Shing Faculty of Medicine, The University of Hong Kong, Hong Kong, China

## Abstract

Lawrence Gostin and colleagues offer a set of priorities for global health preparedness and response for future infectious disease threats.

**Summary Points**

- Four global commissions reviewing the recent Ebola virus disease epidemic response consistently recommended strengthening national health systems, consolidating and strengthening World Health Organization (WHO) emergency and outbreak response activities, and enhancing research and development.
- System-wide accountability is vital to effectively prevent, detect, and respond to future global health emergencies.
- Global leaders (e.g., United Nations, World Health Assembly, G7, and G20) should maintain continuous oversight of global health preparedness, and ensure effective implementation of the Ebola commissions’ key recommendations, including sustainable and scalable financing.

The world is becoming increasingly vulnerable to pandemics resulting from globalization, urbanization, intense human/animal interchange, and climate change. A series of global health crises have emerged since 2000, ranging from Severe Acute Respiratory Syndrome (SARS) and its phylogenetic cousin Middle East Respiratory Syndrome (MERS), to pandemic Influenza A (H1N1), Ebola, and the ongoing Zika virus epidemic. The Ebola epidemic gave rise to four global commissions proposing a bold new agenda for global health preparedness and response for future infectious disease threats [1–7].

The four commissions, listed in chronological order are: 1) the World Health Organization (WHO) Ebola Interim Assessment Panel (WHO Interim Assessment); 2) the Harvard University and the London School of Hygiene & Tropical Medicine’s Independent Panel on the Global Response to Ebola (Harvard/LSHTM); 3) the Commission on a Global Health Risk Framework for the Future (CGHRF) convened by the US National Academy of Medicine; and 4) the United Nations High-Level Panel on the Global Response to Health Crises (UN Panel). In response to critiques of WHO’s performance during the Ebola crisis, the World Health Assembly (WHA) approved an Advisory Group on Reform of WHO’s Work in Outbreaks and Emergencies, which reported in January 2016. The WHA also approved a Review Committee on the International Health Regulations (2005) (IHR), due to report in May [8].

The commissions were established to critically evaluate the national and global response to Ebola and to enhance preparedness to prevent, detect, and respond to future infectious disease threats. Each commission had its own membership and funding described in S1 Table, but had similar mandates to improve global health security. Given the major threat posed by infectious diseases, these panel reports should drive the agendas of the WHA and the G7 Summit in 2016, and global leaders should then maintain heightened oversight of global health preparedness going forward.

Pandemics pose a significant risk to security, economic stability, and development. The CGHRF estimated annualized expected losses from pandemics at $60 billion per year—$6 trillion in the 21^st^ century—yet the global community has significantly underestimated and under-invested in pandemic threats. CGHRF recommended an annual incremental investment of $4.5 billion—65 cents per person—to strengthen global preparedness. This modest investment would provide a major security dividend.

This article focuses on three major reform dimensions—national health systems, global governance, and research and development—and offers a set of priorities drawing on the findings of all four commissions. S1 and S2 Tables compare the four commissions’ reports along these dimensions. To make the world safer, we need robust health systems; an empowered WHO, with strengthened response capacities; a well-funded and planned research and development strategy; and system-wide accountability.

## National Health Systems

**Fig 1:**
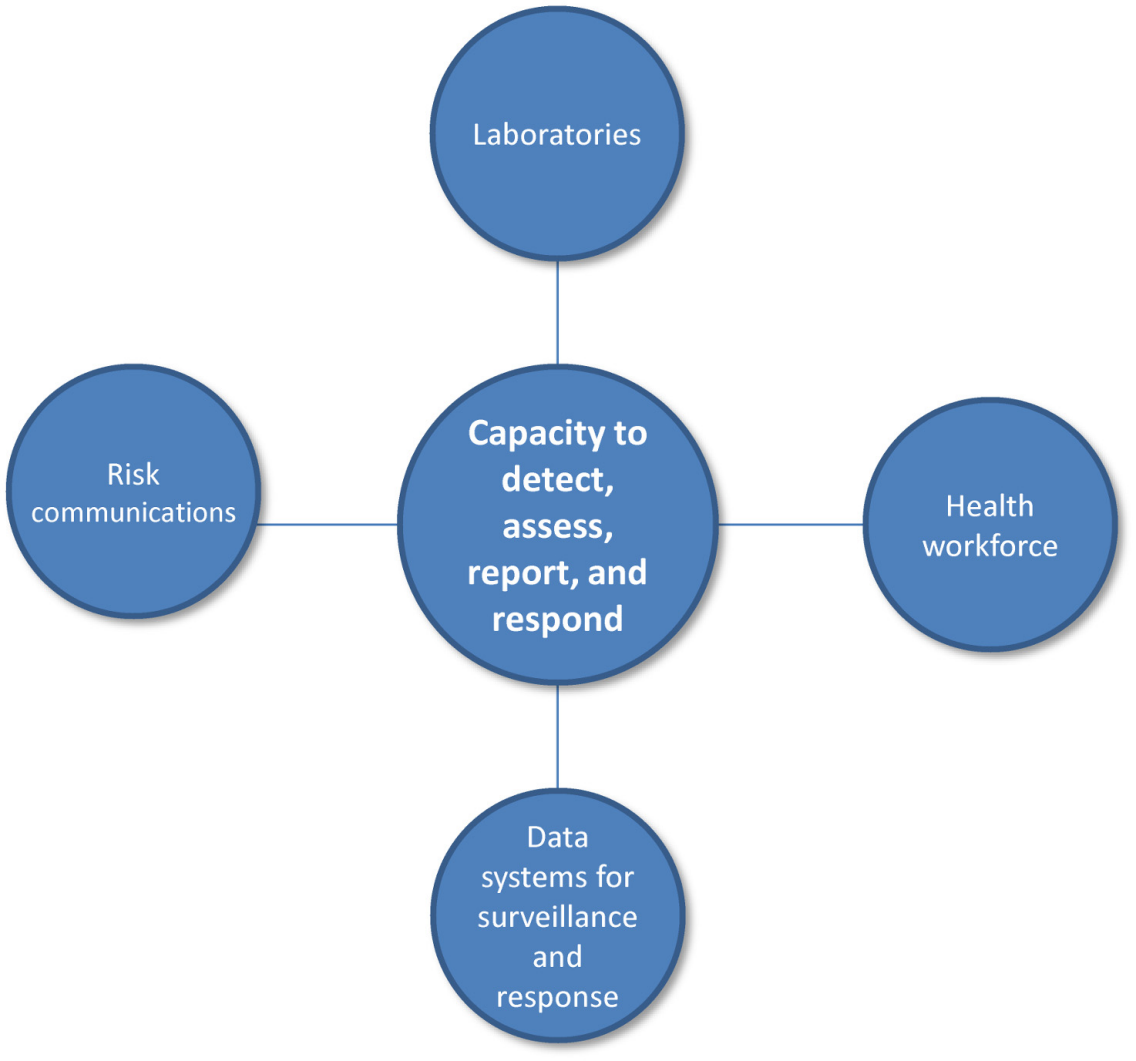
IHR Core Capacities.

Robust and sustainable health systems are an indispensable prerequisite for health security. The IHR—the governing framework for managing infectious disease outbreaks—requires 196 States Parties to develop and maintain core health system capacities to detect, assess, report, and respond to potential public health emergencies of international concern (PHEIC) [9]. Core capacities include a health workforce, laboratories, data systems, and risk communication to identify and contain threats before they cross national borders (Fig 1). The initial target date for establishing these capacities was June 2012 [10].

WHO has traditionally measured national health capacities by allowing states to conduct annual self-assessments. Most states, however, missed the initial 2012 reporting requirement for meeting IHR core capacities and, in 2014, WHO extended the deadline to 2016 for all 81 states that requested extensions. Of the 193 states required to report, only 64 states reported meeting core capacities, representing a compliance rate of just over 30%, while 48 failed to even respond [10]. This low level of compliance for meeting minimum core capacity standards may be an overestimate because self-assessments are unreliable without independent validation.

Table 1 highlights the commissions’ recommendations to develop and assess core capacities. The WHO took an important step in February 2016, developing a Joint External Evaluation Tool to evaluate IHR capacities every 5 years, with national and international subject experts reviewing self-reported data, followed by a country visit and in-depth discussions. Each country’s assessment will be made public, with a color-coding scheme to delineate implementation levels for each capacity [11]. Until governments achieve a high degree of compliance with IHR obligations, however, WHO should plan more frequent external assessments, rather than waiting 5 years between assessments. Participation of stakeholders in the community should also be a critical component of assessments.

**Table 1:** Recommendations from the Four Global Commissions Concerning National Health Systems—Core Capacity Compliance.

	CGHRF	Harvard/LSHTM	UN Panel	WHO Interim Assessment
**Development of IHR Core Capacities**	By mid-2017, all countries should develop and publish plans to achieve and maintain their IHR core capacities. (Rec. B.6)	The global community must agree on a clear plan for national governments to invest domestically in building IHR core capacities. (Rec. 1)	No recommendation.	WHO should create a costed and prioritized plan for all countries to develop IHR core capacities. Financing should be provided in partnership with the World Bank. (Rec. 1)
**Assessment of IHR Core Capacities**	By the end of 2016, WHO should devise a regular, independent, transparent, and objective assessment mechanism for evaluating IHR core capacities. (Rec. B.2) All countries should consent to external assessment. (Rec. B.3) WHO and its member states should agree on precise benchmarks for evaluating core capacities that go beyond standard implementation checklists. (Rec. B.1)	All countries must agree to regular, independent, external assessment of their IHR core capacities. (Rec. 1)	WHO should strengthen its periodic review of compliance with IHR core capacity requirements to ensure that all member states are subject to an independent, field-based assessment at least once every four years on a rotating basis. After an assessment is completed, WHO’s Secretariat should follow up within 3 months with a costed action plan to address any deficiencies. (Rec. 6)	Assessment of IHR core capacities must be based on independently assessed information, validated through some form of peer review or other external assessment. (Rec. 1)
**Deadline for Compliance with IHR Core Capacity Requirements**	By 2020, all countries should be fully compliant with IHR core capacity requirements. (Rec. B.6)	No recommendation.	By 2020, all state parties to the IHR should be in full compliance with the core capacities requirements. (Rec. 1)	No recommendation.

A major weakness of WHO’s new mechanism for monitoring the core capacities of states is that it is voluntary, reflecting state sovereignty concerns. Ensuring compliance with IHR obligations, even with better reporting, requires creative incentives, technical and financial support, and transparency. Table 2 highlights the commissions’ recommendations on financing and incentives towards ensuring countries report their core capacities and meet their minimum IHR obligations. The International Monetary Fund (IMF) could encourage countries to participate in ongoing evaluations by incorporating pandemic preparedness into its evaluation of macroeconomic stability [4]. IMF assessments offer a powerful inducement given their influence over countries’ access to capital. Similarly, the World Bank’s Pandemic Emergency Facility—along with regional development banks—could condition disbursements based on governments meeting IHR obligations.

**Table 2:** Recommendations from the Four Global Commissions Concerning National Health Systems—Core Capacity Financing and Incentives.

	CGHRF	Harvard/LSHTM	UN Panel	WHO Interim Assessment
**Technical & Financial Assistance**	WHO should provide technical support to countries to fill gaps in IHR core capacities. WHO’s Centre for Health Emergency Preparedness & Response support should coordinate this support. (Rec. B.7) The World Bank should convene its development partners to provide financial assistance to lower-middle and low-income countries. (Rec. B.9)	Adequate external support should be provided to supplement efforts to build IHR core capacities in poorer countries. (Rec. 1)	The WHO Director-General (DG) should lead efforts to mobilize both financial and technical support to build IHR core capacities, in partnership with the World Bank, donors, foundations, and the private sector. (Rec. 17)	No recommendation.
**Incentives for Participating in Core Capacity Assessments**	The World Bank, bilateral donors, and multilateral donors should make funding of health systems contingent on the participation of the recipient in the external assessment process. (Rec. B.4) The IMF should include pandemic preparedness in its assessments of individual countries. (Rec. B.5)	No recommendation.	No recommendation.	No recommendation.

Political leaders are more likely to cooperate if they view external evaluations as a pathway to funding and technical support. Consequently, WHO should not simply give countries a pass/fail grade; rather, it should constructively partner with governments. Core capacity financing, however, requires the international community to close an investment gap of $3.4 billion per year [4]. WHO and the World Bank should develop a financial plan, with targets for national and international contributions. Additional financing mechanisms could be modeled on the Global Fund, with the World Bank and WHO hosting periodic donor investment and replenishment conferences.

The Global Health Security Agenda (GHSA), a partnership initiated by the United States encompassing nearly 50 countries, which was set up to prevent, detect, and respond to future infectious disease outbreaks, could offer a model for strengthening health systems [12]. GHSA, with >$1 billion in funding, has developed “Action Packages,” where priority technical areas are identified, with each encompassing a target and action items, along with baseline assessment, planning, and monitoring activities, and capabilities evaluated through a peer assessment process [13]. GHSA, however, formally stands outside the IHR framework, thus lacking the international legitimacy of a WHO-led process.

The minimum core capacities set out by the IHR on their own are insufficient to respond to public health threats and emergencies, however, as highlighted by the commissions’ recommendations in Table 3. Effective primary care and public health systems that underpin inclusive, high-quality universal health coverage (UHC) are also required to manage outbreaks and meet a broad range of health needs to ensure the right to health. Fast-spreading novel infections are diverse, demanding resilient health systems. As outbreaks stretch existing resources, resilient systems that are designed to ensure surge capacity in health emergencies are needed.

**Table 3:** Recommendations from the Four Global Commissions Concerning National Health Systems—Key Components.

	CGHRF	Harvard/LSHTM	UN Panel	WHO Interim Assessment
**Training of Health Professionals**	No recommendation.	No recommendation.	Governments should increase spending on training health professionals, particularly community health workers, who are most familiar with the local culture. (Rec. 2)	No recommendation.
**Community Engagement**	Governments and WHO should increase engagement with non-state actors, including community leaders, civil society organizations, the private sector, and the media. (Rec. C.6)	No recommendation.	Governments and responders must streamline their community engagement to promote local ownership and trust. (Rec. 3)	WHO and its partners must ensure that appropriate community engagement is a core function when managing a health emergency. (Rec. 15)
**Gender Inclusion**	No recommendation.	No recommendation.	Efforts to improve outbreak preparedness and response must include women at all levels of planning and operations and must take women’s needs into account, as they most often act as primary care-givers. (Rec. 4)	No recommendation.

The UN Sustainable Development Goals (SDGs) expressly encompass infectious disease outbreaks and set a target for UHC by 2030 [14]. Many of the commissions’ recommendations fall within the SDGs’ framework as described in S2 Table. The SDGs, in supporting universal health systems, stress health equity, which is also vital because outbreaks often emerge in marginalized communities and then rapidly spread.

Achieving resilient health systems is a shared national and global responsibility. By 2017, every government should develop and publish concrete plans to achieve IHR core capacities by 2020. By the end of the following decade, all nations should achieve the SDG target of health coverage for all.

## Global Governance for Health

The commissions’ reports reflecting on the Ebola epidemic echoed a crucial point made by the IHR Review Committee on the response to the H1N1 pandemic in its 2011 report—“the world is ill-prepared for a severe pandemic or for any similarly global, sustained and threatening public health emergency” [15]. At an international level, the commissions’ reports focused on reforms for WHO and the UN System, but also discussed the role of the World Bank and World Trade Organization (WTO) (Table 4).

**Table 4:** Recommendations from the Four Global Commissions Concerning Global Governance—International Coordination.

	CGHRF	Harvard/LSHTM	UN Panel	WHO Interim Assessment
**Pandemic Emergency Financing Facility**	By the end of 2016, the World Bank should establish a Pandemic Emergency Financing Facility as a rapidly deployable source of funds to support pandemic response. (Rec. C.9)	No recommendation.	The World Bank should expeditiously operationalize its Pandemic Emergency Financing Facility. (Rec. 21)	No recommendation.
**Coordination Between WHO and WTO on Trade and Health**	No recommendation.	No recommendation.	The WTO and WHO should convene a joint commission to ensure that their respective legal frameworks apply consistent standards with respect to trade restrictions imposed for public health reasons. (Rec. 24)	No recommendation.

## World Health Organization

If national health systems are the foundation for global health security, WHO is at the apex [16]. Yet WHO faces a crisis of confidence, with major critiques of its performance during Ebola. The Organization had previously cut nearly two-thirds of its emergency response unit; delayed four-and-a-half months before declaring a PHEIC; and lacked the governance needed to coordinate multiple stakeholders, including its regional and country offices [17]. As featured in the recommendations in Table 5, the commissions called for emergency preparedness and response to become “a core part of WHO’s mandate, positioning itself as an operational organization” [8].

**Table 5:** Recommendations from the Four Global Commissions Concerning Global Governance—WHO Emergency Operations and Response Reform.

	CGHRF	Harvard/LSHTM	UN Panel	WHO Interim Assessment
**Independent Centre for Preparedness & Response**	WHO should create a Centre for Health Emergency Preparedness & Response (CHEPR), governed by an independent Technical Governing Board, to coordinate global outbreak preparedness and response. (Rec. C.1)	WHO should create a unified Centre for Emergency Preparedness & Response with clear responsibility, adequate capacity, and strong lines of accountability. (Rec. 3)	WHO’s Program for Outbreaks & Emergency Management should be converted into a Centre for Emergency Preparedness & Response (CEPR) with unified command and control authority. (Rec. 7)	WHO should establish a Centre for Emergency Preparedness & Response that integrates its outbreak control and humanitarian functions. (Rec. 11) An independent board should oversee the Centre and provide an annual global health security report to the WHA and UN GA. (Rec. 12)
**Create Contingency Fund for Rapid Response**	By the end of 2016, WHO should create a sustainable contingency fund of US$100 million to support rapid deployment of emergency response capabilities. (Rec. C.3)	No recommendation.	WHO should establish a contingency fund for emergency response, managed by the CEPR. Member States should provide at least US$300 million in financing. (Rec. 20)	Member States and partners should contribute to a contingency fund in support of outbreak response, with a minimum target capitalization of US$100 million. (Rec. 8)
**Communications & Outbreak Monitoring**	WHO should generate a high-priority “watch list” of outbreaks, released daily to national focal points and weekly to the public. (Rec. C.7)	Responsibility for declaring a PHEIC should be delegated to a transparent and politically protected WHO standing committee. (Rec. 4)	WHO must re-establish itself as the authoritative body for health emergencies, capable of rapidly and accurately informing governments and the public about the severity and extent of an outbreak. (Rec. 14)	The IHR Review Committee should consider the creation of an intermediate level of emergency to alert the international community at an earlier stage of a health crisis before it becomes a global threat. (Rec. 5)

The commissions also unanimously recommended that WHO create a Centre for Emergency Preparedness and Response (CEPR), integrating and strengthening all its preparedness, response, and humanitarian activities. The Centre would have a clear mandate, separate funding streams, and strong lines of accountability. An Executive Director (at the Deputy Director-General level) would lead the Centre, reporting to an independent oversight body. CGHRF recommended a Technical Governing Board comprised of independent experts to hold the CEPR accountable.

Member States have resisted providing the funding needed for WHO to fulfill its global mandate, and most existing funding is earmarked. The absence of sustainable and scalable financing could diminish WHO’s capacity to manage future outbreaks. Table 6 highlights the commissions’ recommendations regarding WHO’s ongoing reforms. As WHO’s emergency operational capacities are upgraded, states must not cut funding for its other core activities, including noncommunicable diseases, injuries, and mental health—the Harvard/LSHTM panel recommended “its functions should be far more circumscribed” [3].

**Table 6:** Recommendations from the Four Global Commissions Concerning Global Governance—Ongoing WHO Reform.

	CGHRF	Harvard/LSHTM	UN Panel	WHO Interim Assessment
**Increase Member State Assessed Contributions**	In May 2016, the WHA should agree to appropriately increase WHO Member States’ core contributions in order to provide sustainable financing for the CHEPR. (Rec. C.2)	No recommendation.	Member States should increase contributions to the WHO budget by at least 10 percent. Ten percent of all voluntary contributions to the WHO budget should be earmarked to support the CEPR. (Rec. 18 & 19)	At the 2016 WHA meeting, Member States should consider moving from zero nominal growth in assessed contributions to an increase of 5 percent. (Rec. 7)
**Operations & Governance**	No recommendation.	WHO should focus on its core functions and implement good governance reforms. (Rec. 9 & 10)	No recommendation.	WHO must develop an organizational culture for emergency preparedness and response. (Rec. 10)
**Integrating Regional & Sub-Regional Networks**	WHO should strengthen its linkages with regional and sub-regional networks to enhance mutual support and trust, to promote sharing of information and laboratory resources, and facilitate joint outbreak investigations among neighboring countries. (Rec. C.5)	No recommendation.	WHO should support the efforts of regional and sub-regional organizations to develop and strengthen their standing capacities to monitor, prevent, and respond to health crises. (Rec. 5)	No recommendation.
**Country-Specific Staffing & Planning**	WHO should work with the UN Secretary-General (SG) and other UN bodies to develop strategies for sustaining health system capabilities and infrastructure in failed states and in war zones. (Rec. B.10)	No recommendation.	No recommendation.	WHO must take local circumstances into account when staffing its country offices. WHO representatives must have the full support of regional directors and the WHO DG if challenged by national governments. (Rec. 13)
**Renegotiate the Pandemic Influenza Preparedness Framework**	No recommendation.	No recommendation.	WHO member states should re-negotiate the Pandemic Influenza Preparedness Framework to include other novel pathogens and make it legally binding. (Rec. 15)	No recommendation.
**Holding National Governments Accountable**	The WHA should agree on new mechanisms for holding national governments publicly accountable for their performance under the IHR, including protocols to prevent delays in reporting and protocols for avoiding unnecessary restrictions on travel and trade. (Rec. C.8)	WHO should publicly commend countries that rapidly share information and publish lists of countries that delay reporting. Funders should create incentives for early reporting by disbursing emergency assistance rapidly. WHO must confront governments that implement travel and trade restrictions without justification. (Rec. 2)	The IHR Review Committee should develop mechanisms to rapidly address unilateral action by member states in contravention of temporary recommendations issued by WHO as part of a PHEIC announcement. (Rec. 23)	The IHR Review Committee should consider financial incentives for early reporting, including insurance to mitigate adverse economic effects. The Committee also should consider financial disincentives to discourage countries from restricting trade and travel beyond measures recommended by WHO. (Rec. 3 & 4)

In March 2016, the Secretariat launched WHO’s Health Emergencies Programme to assure “cross-organizational standards and rapid decision-making in health emergency operations” [7]. The Programme’s development was influenced by the Advisory Group on Reform of WHO’s Work in Outbreaks and Emergencies, whose recommendations are highlighted in Table 7. Whether the Programme meets the panels’ standards for a quasi-independent Centre of high quality and accountability remains to be determined. Currently, the Programme has no sustained funding or independent governance. However, the Director-General is establishing an Independent Oversight and Advisory Committee to monitor and oversee the Programme’s performance.

**Table 7:** Recommendations of the WHO Advisory Group.

RECOMMENDATIONS	Advisory Group on Reform of WHO’s Work in Outbreaks and Emergencies
**Independent Center for Preparedness & Response**	WHO should establish a centrally-managed global Programme for Outbreaks and Emergencies to integrate the functions of units at all three organizational levels (country, regional, and headquarters) that work on risk analysis and assessment and on preparedness and response. The Programme should have one budget and a single workforce reporting to the WHO DG. (Rec. 2)
**Lines of Authority For Incident Management**	The Programme should be headed by an Executive Director reporting to the WHO DG, who will remain ultimately accountable for incident management. When WHO declares a global health emergency, the Executive Director should appoint an Incident Manager to coordinate WHO’s response. Incident Managers, heads of Country Offices, and Regional Directors should establish good working relationships and be held accountable. To maintain flexibility, lines of authority for incident management should shift from their default positions, depending on the severity of the outbreak or emergency. (Rec. 3)
**Strategic Collaboration Between WHO and Health Partners at the National Level**	As a standard component of its operational planning, WHO should undertake a stakeholder analysis at the national level to identify potential health partners. This analysis should look beyond traditional government ministry partners to include private sector actors, civil society organizations, and faith-based groups. WHO should review the appropriate partners for co-leadership of Health Clusters at the national level and work with partners to build a dedicated capacity for coordination, planning, information management, and communications. WHO should integrate the capacities of its Health Cluster partners in its emergency preparedness and planning. (Rec. 4)
**Reform of WHO Operations & Governance**	WHO must urgently develop new business processes governing procurement and logistics, as well as the rapid deployment of human and financial resources, during outbreaks and emergencies. These processes should be tailored to support the Programme for Outbreaks and Emergencies and should not be the same as those used for WHO’s ordinary business operations. Benchmarks should be established to assess whether these new processes are timely and effectively implemented. (Rec. 5)
**Increase Member State Assessed Contributions**	WHO should make a clear distinction between the resources necessary to support the baseline capacity of the Programme for Outbreaks and Emergencies and funding needed to support specific emergency operations. Predictable and reliable financing streams, including assessed contributions from member states, should fund the baseline capacity of the Programme. Member states must be willing to provide the resources for the Programme to meet expectations. (Rec. 6(a), (b))
**Contingency Fund For Rapid Response**	For programmatic funding to support emergency operations, WHO should maximize its use of existing funding mechanisms, such as the Central Emergency Response Fund managed by the Emergency Relief Coordinator on behalf of the UN SG, and actively seek the full capitalization (US $100 million) of the new Contingency Fund for Rapid Response. (Rec. 6(c))
**Resource Mobilization& Political Engagement**	WHO should exercise transparency in resource management by showing how existing resources can be used more efficiently, by clearly articulating the linkages between resources and specific outcomes, by identifying benchmarks to assess progress on deliverable outcomes, and by rigorously tracking its expenditures. WHO should communicate a broader vision of its role that explains how investing in the Programme for Outbreaks and Emergencies will be cost-effective. WHO also should more narrowly tailor its political engagement, soliciting input from donors and stakeholders. (Rec. 7)
**Accountability & Oversight**	The WHO DG should establish an external, independent oversight body to monitor the performance of the Programme for Outbreaks and Emergencies using benchmarks established for this purpose. Members of the oversight body should have technical expertise in areas relevant to the operation of the Programme. The membership should be multi-sectoral and may be drawn from member states, donors, NGOs, civil society, the private sector, and the UN system. (Rec. 8)

## The United Nations System

When a health crisis escalates to a humanitarian disaster, even a well-resourced WHO will be unable to galvanize political will and coordinate a broader response, requiring operational control to shift to the United Nations. Table 8 highlights the commissions’ recommendations on the UN’s role and responsibilities during a health crisis. WHO should advise the UN Emergency Relief Coordinator to make this determination based on criteria for a Level 3 emergency (the highest level), including the epidemic’s scale, economic toll, and political destabilization [18]. The UN Inter-Agency Standing Committee, “the primary mechanism for inter-agency coordination of humanitarian assistance,” [19] would establish procedures for UN inter-agency coordination [6].

**Table 8:** Recommendations from the Four Global Commissions Concerning Global Governance—Ongoing UN Reform.

	CGHRF	Harvard/LSHTM	UN Panel	WHO Interim Assessment
**Improve Coordination and Cooperation**	The UN and WHO should establish clear mechanisms for coordination and escalation in health crises, including those that become part of broader humanitarian crises that require the mobilization of the entire UN system. (Rec. C.4)	The UN SC should establish a Global Health Committee to elevate the level of attention paid to public health issues and to mobilize political leadership. (Rec. 8)	In the event of a Grade 2 or Grade 3 outbreak not already classified as a humanitarian emergency, a clear line of command should be activated throughout the UN system. (Rec. 8) The SG should integrate the UN’s health and humanitarian crisis trigger systems. (Rec. 9)	WHO should coordinate its emergency grades and its criteria for declaring a PHEIC with the emergency levels applied in the broader humanitarian system to facilitate better inter-agency cooperation. (Rec. 18)
**System-Wide Accountability**	No recommendation.	An independent UN Accountability Commission, reporting to WHA and the UN SC, should be established to perform system-wide assessments of worldwide responses to disease outbreaks. (Rec. 5)	The UN GA should create a High-Level Council on Global Public Health Crises to track the implementation of reforms and to monitor political and other non-health issues that may affect prevention and preparedness. (Rec. 26)	The UN should put global health issues at the top of its security agenda and refer significant threats to the SC. (Rec. 6) When a crisis escalates, the UN SG should consider the appointment of Special Representative or Special Envoy. (Rec. 21)

A UN Security Council resolution would elevate a crisis to the top tier of the global agenda. As binding international law, a Council resolution would be more effective in mobilizing resources, sustaining political will, and securing state compliance with WHO recommendations. The Secretary-General could also appoint a special envoy or establish a mission to implement Security Council directives [6].

It is also important to ensure ongoing UN engagement during inter-crisis periods. The UN High-Level Panel recommended the General Assembly form a Council on Global Health Crises, while the Harvard/LSHTM panel proposed a standing Global Health Committee within the Security Council. Creating a standing presence in a UN organ and, where necessary, declaring a Level 3 Emergency would raise the public and political profile of global health security in ways WHO has been unable to achieve.

## Research and Development (R&D)

The Ebola and Zika epidemics revealed systemic deficiencies in R&D for diagnostic tests, vaccines, and therapies. The paucity of medical technologies stem primarily from low commercial priority, limited funding, and practical challenges of conducting human trials for episodic infections. Yet medical countermeasures are vital to contain outbreaks and minimize their impact.

As highlighted by the recommendations in Table 9, WHO has a central role in establishing the normative framework for R&D including priority setting, accelerating trial design and administration, regulatory pathways, and equitable access. The Organization spearheaded an international effort for an Ebola vaccine. More recently, WHO identified eight pathogens as research priorities, but at a high level of generality [20]. In March 2016, WHO published more granular priorities for Zika, including vector control [21]. Vaccine platform technologies may be able to accelerate the development of a vaccine for the Zika virus, as well as other pathogens. Yet, even with expedited vaccine development, inadequate access to existing vaccines still persists. For example, Yellow Fever vaccine stockpiles during a recent outbreak have been exhausted. CGHRF recommended an independent Pandemic Product Development Committee to mobilize resources, coordinate public/private actors, and create a strategic R&D plan.

**Table 9:** Recommendations from the Four Global Commissions Concerning Research and Development—R&D Acceleration.

	CGHRF	Harvard/LSHTM	UN Panel	WHO Interim Assessment
**WHO’s Role in R&D**	WHO should establish an independent Pandemic Product Development Committee (PPDC), accountable to the Technical Governing Board, to spearhead its efforts to galvanize and prioritize R&D. (Rec. D.1)	No recommendation.	WHO should coordinate the prioritization of R&D efforts to combat neglected diseases that pose the greatest risk of turning into global health crises. (Rec. 13)	WHO should play a central convening role in R&D efforts during future emergencies, including efforts to accelerate the development of appropriate diagnostics, vaccines, therapeutics, and information technology. (Rec. 16)
**Accelerating R&D**	By the end of 2016, the PPDC should convene national regulators, industry stakeholders, and research organizations to accelerate R&D by promoting regulatory convergence; the pre-approval of clinical trial designs; mechanisms to manage intellectual property, data sharing, and product liability; and efforts to expedite vaccine manufacture, stockpiling, and distribution. (Rec. D.3)	Governments, researchers, private industry, and non-governmental organizations must develop a framework of norms and rules operating during and between outbreaks to enable and accelerate research, govern the conduct of research, and ensure access to the benefits of research. (Rec. 6)	No recommendation.	No recommendation.
**R&D in Developing Countries**	No recommendation.	No recommendation.	WHO should lead efforts to assist developing countries in building research and manufacturing capacities for vaccines, therapeutics, and diagnostics, including through South-South cooperation. (Rec. 16)	No recommendation.

CGHRF, joined by other panels, urged $1 billion incremental funding per year from combined governmental and private sources to jumpstart research innovations (Table 10). Beyond medical technologies, targeted investments would facilitate manufacturing capacity in lower-income countries, improve personal protective equipment effectiveness, enhance information systems, and integrate the study of zoonotic pathogens into ongoing research. The commissions placed a premium on scientifically and ethically rigorous trial designs and research participants’ rights.

**Table 10:** Recommendations from the Four Global Commissions Concerning Research and Development—Financing.

	CGHRF	Harvard/LSHTM	UN Panel	WHO Interim Assessment
**Global Financing Facility for Infectious Diseases with Pandemic Potential**	The PPDC should work with global R&D stakeholders to catalyze the commitment of US$1 billion per annum to fund a portfolio of projects to develop drugs, vaccines, diagnostics, protective equipment, and medical devices to combat communicable diseases. (Rec. D.2)	The UN SG and WHO DG should convene a summit of public, private, and non-profit research funders to establish a global financing facility for the development of outbreak-relevant drugs, vaccines, diagnostics, and supplies for which commercial incentives are insufficient. (Rec. 7)	WHO should oversee an international fund of at least US$1 billion per annum to support R&D of vaccines, therapeutics, and rapid diagnostics for communicable diseases neglected by the commercial market. (Rec. 22)	No recommendation.

Research requires sharing biological materials, but governments sometimes delay. Concerned about affordable access to the fruits of research, states have claimed sovereignty over viruses under the 2010 Nagoya Protocol to the Convention on Biological Diversity. The Pandemic Influenza Preparedness (PIP) Framework—negotiated by WHO—requires states to share biological materials and pharmaceutical companies to provide reciprocal benefits, such as a share of the resulting drugs or vaccines. The PIP Framework, however, applies only to pandemic influenza and not to other novel pathogens. The UN High-Level Panel recommended that WHO re-negotiate the PIP Framework to expand its scope and make it legally binding.

## System-wide Accountability

**Fig 2:**
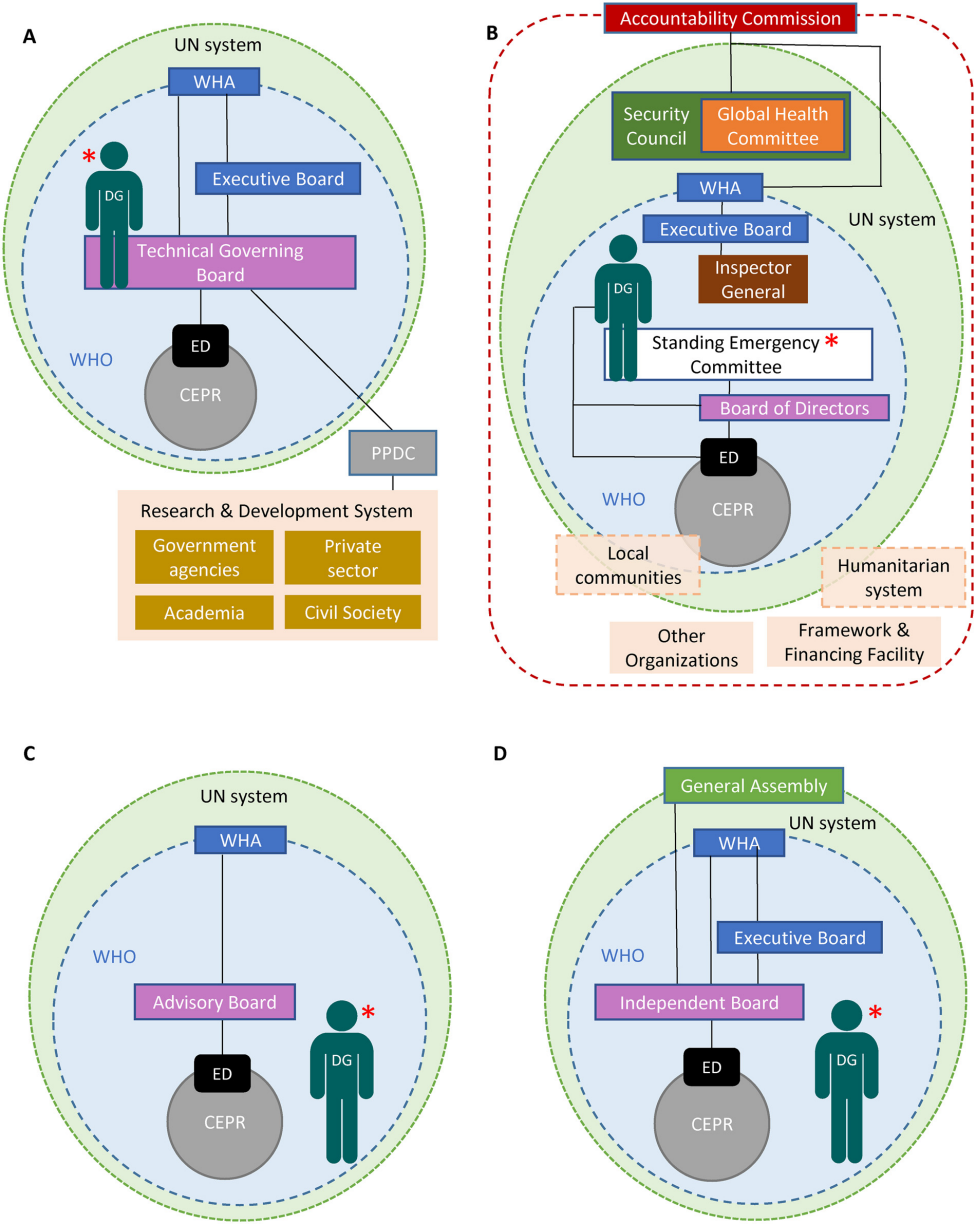
Accountability frameworks proposed by the four commissions during inter-emergencies period. (A) Commission on a Global Health Risk Framework for the Future, (B) Harvard-London School of Hygiene & Tropical Medicine’s Independent Panel on the Global Response to Ebola, (C) United Nations High-Level Panel on the Global Response to Health Crises, (D) World Health Organization Ebola Interim Assessment Panel. * denotes individual/body with responsibility for declaring a Public Health Emergency of International Concern (PHEIC). CEPR = Centre for Emergency Preparedness and Response, DG = Director-General, ED = Executive Director, PPDC = Pandemic Product Development Committee, SG = Secretary-General, UN = United Nations, WHA = World Health Assembly, WHO = World Health Organization.

**Fig 3:**
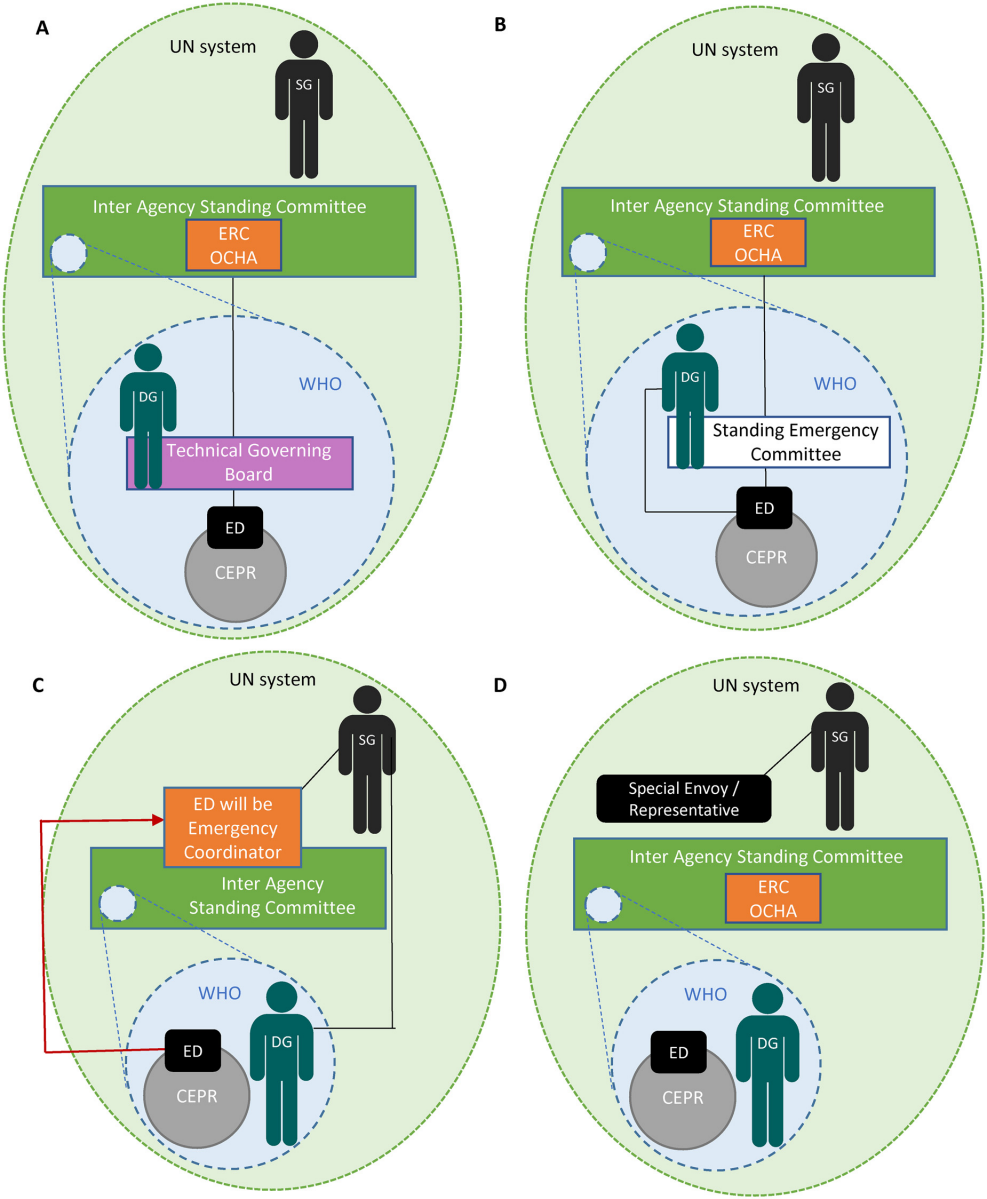
Changes to the accountability frameworks during a PHEIC that has turned into a humanitarian crisis. (A) Commission on a Global Health Risk Framework for the Future, (B) Harvard-London School of Hygiene & Tropical Medicine’s Independent Panel on the Global Response to Ebola, (C) United Nations High-Level Panel on the Global Response to Health Crises, (D) World Health Organization Ebola Interim Assessment Panel. CEPR = Centre for Emergency Preparedness and Response, DG = Director-General, ED = Executive Director, ERC = Emergency Response Coordinator, OCHA = Office for the Coordination of Humanitarian Affairs, SG = Secretary-General, UN = United Nations, WHO = World Health Organization.

Implementing the commissions’ bold agenda requires system-wide accountability. Figs 2 and 3 demonstrate the commissions’ suggested accountability frameworks during both inter-crisis and global health emergency periods. All frameworks would require continual communication and inter-agency collaboration, as well as partnerships with multiple stakeholders. The UN High-Level Panel called for a Summit on Global Public Health Crises in 2018 to assess implementation. The Harvard/LSHTM Panel went further, proposing a UN Accountability Commission to oversee the full range of actors [3].

The commissions proposed greater WHO accountability, including independent oversight of a future CEPR. To conform with WHO’s Constitution, accountability would reside in the Executive Board to which the new Centre would report, with full transparency. The Independent Oversight and Advisory Committee of the newly established WHO Health Emergencies Programme will report to the Executive Board, but ultimate authority rests with the Director-General for WHO’s work in health emergencies.

Holding sovereign governments accountable poses the greatest challenge. States have sometimes failed to promptly report potential PHEICs or share crucial health information. Many states have erected unnecessary travel and trade restrictions or infringed on human rights. The commissions unanimously recommended that the Director-General publicly name governments that fail to act as responsible global citizens [22]. Beyond transparency, it may be possible to coax states’ compliance with their IHR obligations through skilled diplomacy and incentives.

## Political Leadership

The commissions’ proposals are ambitious, with action needed everywhere from civil society and research laboratories to Geneva and national capitals. Political attention to global health security can no longer be episodic, limited to when an epidemic strikes. Political leadership must be sustained by standing agendas on health security at the WHA, G7, G20, and Security Council, with the United Nations overseeing crucial reforms. The commissions have defined a path forward. It would be a reckless disregard for human life and security to resist vital reforms.

## Supporting Information

S1 Table Four Global Commissions in the Wake of Ebola. (DOCX)

S2 Table Recommendations from the Four Global Commissions according to the Sustainable Development Goals framework. (DOCX)
