# Supplementary material for: Toward a Common Secure Future: Four Global Commissions in the Wake of Ebola
Source: PLoS Med. 2016 May 19;13(5):e1002042. doi: 10.1371/journal.pmed.1002042 (PMC4873000; doi:10.1371/journal.pmed.1002042)
Supplement: S1 Table — (DOCX) [file pmed.1002042.s001.docx]

**Supplementary Table 1. Four Global Commissions in the Wake of Ebola**

| Coordinating Secretariat | |
| --- | --- |
| *CGHRF* | National Academy of Medicine |
| *Harvard/LSHTM* | Harvard Global Health Institute, London School of Hygiene & Tropical Medicine |
| *UN Panel* | United Nations Secretary-General’s Office |
| *WHO Interim Assessment* | World Health Organization |
| Funder(s) | |
| *CGHRF* | Paul G. Allen Family Foundation, Ford Foundation, Bill & Melinda Gates Foundation, Ming Wai Lau, Gordon and Betty Moore Foundation, Rockefeller Foundation, USAID, Wellcome Trust |
| *Harvard/LSHTM* | Rockefeller Foundation |
| *UN Panel* | United Nations (particularly Germany and Norway) |
| *WHO Interim Assessment* | World Health Organization |
| Commissioners (Affiliation)  * Indicates individual was a member of more than one commission | |
| *CGHRF* | - **Peter Sands (Chair)** Harvard Kennedy School; - **Ximena Aguilera** Universidad del Desarrollo, Chile - **Irene Akua Agyepong,** Ghana Health Service - **Yvette Chesson Wureh**, Angie Brooks International Centre for Women’s Empowerment, Leadership Development, International Peace & Security - **Paul Farmer**, Harvard Medical School - **Maria Freire**, Foundation for the National Institutes of Health - **Julio Frenk***, University of Miami - **Lawrence Gostin***, O’Neill Institute for National and Global Health Law, Georgetown University - **Gabriel Leung***, The University of Hong Kong - **Francis Omaswa**, African Center for Global Health and Social Transformation - **Melissa Parker**, London School of Hygiene & Tropical Medicine - **Sujatha Rao**, Ministry of Health and Family Welfare of India - **Daniel Ryan**, Swiss Re - **Oyewale Tomori (Vice Chair)**, Nigeria Academy of Sciences - **Jeanette Vega**, Chilean National Health Fund - **Suwit Wibulpolprasert**, Ministry of Public Health, Thailand - **Tadataka Yamada**, Frazier Life Sciences |
| *Harvard/LSHTM* | - **Peter Piot (Chair),** London School of Hygiene & Tropical Medicine - **Chelsea Clinton**, Bill, Hillary & Chelsea Clinton Foundation - **Sophie Delaunay,** Médecins Sans Frontières - **Valnora Edwin,** Campaign for Good Governance - **Mosoka Fallah,** Action Contre La Faim International - **David Fidler,** Indiana University - **Laurie Garrett,** Council on Foreign Relations - **Eric Goosby,** University of California, San Francisco - **Lawrence Gostin*,** O’Neill Institute for National and Global Health Law, Georgetown University - **David Heymann,** Chatham House - **Ashish Jha (Co-Chair),** Harvard Global Health Institute - **Kelley Lee,** Simon Fraser University - **Gabriel Leung*,** The University of Hong Kong - **Suerie Moon (Study Director),** Harvard Global Health Institute - **Steve Morrison,** Center for Strategic and International Studies - **Muhammad Pate (Co-Chair),** Duke University - **Jorge Saavedra,** AIDS Healthcare Foundation - **Devi Sridhar (Co-chair),** University of Edinburgh - **Marcel Tanner,** Swiss Tropical & Public Health Institute |
| *UN Panel* | - **Jakaya Mrisho Kikwete (Chair),** Former President, Tanzania - **Micheline Calmy-Rey,** Former President, Switzerland - **Celso Amorim**, Diplomat, Brazil - **R. M. Marty M. Natalegawa,** Diplomat, Indonesia - **Joy Phumaphi.** African Leaders Malaria Alliance - **Rajiv Shah,** Georgetown University |
| *WHO Interim Assessment* | - **Barbara Stocking (Chair),** Cambridge University - **Carmencita Alberto-Banatin,** Independent advisor, Philippines - **Julio Frenk*,** University of Miami - **Ilona Kickbusch,** Graduate Institute of International and Development Studies, Geneva - **Jean-Jacques Muyembe-Tamfun,** National Institute for Biomedical Research, Democratic Republic of the Congo. - **Faisal Shuaib,** National Ebola Emergency Operations Center, Nigeria |
