# Supplementary material for: Toward a Common Secure Future: Four Global Commissions in the Wake of Ebola
Source: PLoS Med. 2016 May 19;13(5):e1002042. doi: 10.1371/journal.pmed.1002042 (PMC4873000; doi:10.1371/journal.pmed.1002042)
Supplement: S2 Table — (DOCX) [file pmed.1002042.s002.docx]

**S2 Table. Recommendations from the Four Global Commissions according to the Sustainable Development Goals framework**

| **Goal 3: Ensure healthy lives and promote well-being for all at all ages** | |
| --- | --- |
| *CGHRF* | No recommendation. |
| *Harvard/LSHTM* | No recommendation. |
| *UN Panel* | - The international community must fulfill the commitments towards the SDGs, with a particular emphasis on health-sector goals. (Rec. 10) - Partners sustain their Official Development Assistance to health and direct a greater percentage to strengthening health systems under an agreed-upon government-led plan. (Rec. 11) |
| *WHO Interim Assessment* | No recommendation. |
| **Goal 3.8: Achieve universal health coverage, including financial risk protection, access to quality essential health-care services and access to safe, effective, quality and affordable essential medicines and vaccines for all** | |
| *CGHRF* | No recommendation. |
| *Harvard/LSHTM* | No recommendation. |
| *UN Panel* | - WHO works closely with development actors to ensure that development programming supports health systems and thereby helps improve universal and equitable access to quality health. (Rec. 12) - Urgent measures are taken to ensure universal access to and affordability of medicines, vaccines and other life-saving products. (Rec. 14) |
| *WHO Interim Assessment* | No recommendation. |
| **Goal 3.b: Support the research and development of vaccines and medicines for the communicable and non-communicable diseases that primarily affect developing countries, provide access to affordable essential medicines and vaccines, in accordance with the Doha Declaration on the TRIPS Agreement and Public Health, which affirms the right of developing countries to use to the full the provisions in the Agreement on Trade-Related Aspects of Intellectual Property Rights regarding flexibilities to protect public health, and, in particular, provide access to medicines for all** | |
| *CGHRF* | - WHO should establish an independent PPDC, accountable to the TGB, to galvanize acceleration of relevant R&D, define priorities, and mobilize and allocate resources. (Rec. D1) - WHO should work with global R&D stakeholders to catalyze the commitment of $1 billion/yr to maintain a portfolio of projects coordinated by the PPDC. (Rec. D2) - PPDC should convene regulatory agencies, industry stakeholders, and research organizations to commit to the following actions:   - Adopting R&D approaches during crises that maintain consistently high scientific standards.   - Defining protocols and approaches to engage local scientists and community members in the conduct of research.   - Agreeing on ways to expedite medical product approval, manufacture, and distribution. (Rec. D3) |
| *Harvard/LSHTM* | - Develop a framework of rules to enable, govern, and ensure access to the benefits of research. (Rec. 6) - Establish a global facility to finance, accelerate, and prioritise research and development. (Rec. 7) |
| *UN Panel* | - WHO coordinates the prioritization of global R&D efforts for neglected diseases that pose the greatest threat of turning into health crises. (Rec. 13) - WHO convenes its Member States to re-negotiate the Pandemic Influenza Preparedness Framework with a view to including other novel pathogens, making it legally binding, and achieving an appropriate balance between obligations and benefits, in accordance with the principles of the 2010 Nagoya Protocol to the Convention on Biological Diversity. (Rec. 15) - WHO leads efforts to assist developing countries in building research and manufacturing capacities for vaccines, therapeutics and diagnostics, including through South-South cooperation. (Rec. 16) - WHO oversees the establishment and management of an international fund of at least $1 billion per annum to support R&D of vaccines, therapeutics and rapid diagnostics for neglected communicable diseases. (Rec. 22) |
| *WHO Interim Assessment* | - WHO should play a central convening role in research and development efforts in future emergencies, including the acceleration of the development of appropriate diagnostics, vaccines, therapeutics and medical and information technology. (Rec. 16) |
| **Goal 3.c: Substantially increase health financing and the recruitment, development, training and retention of the health workforce in developing countries, especially in least developed countries and small island developing States** | |
| *CGHRF* | No recommendation. |
| *Harvard/LSHTM* | No recommendation. |
| *UN Panel* | - Governments increase investment in the training of health professionals and establish Community Health Workers (CHWs) systems that are appropriate to country circumstances. (Rec. 2) |
| *WHO Interim Assessment* | No recommendation. |
| **Goal 3.d: Strengthen the capacity of all countries, in particular developing countries, for early warning, risk reduction and management of national and global health risks** | |
| *CGHRF* | - WHO and member states should develop an agreed-on, precise definition and benchmarks for national core capabilities, and functioning, based on IHR and other efforts (GHSA and OIE Terrestrial Animal Health Code). (Rec. B1) - WHO should devise a regular, independent, transparent, and objective assessment mechanism to evaluate country performance against benchmarks defined in B.1. (Rec. B2) - All countries should commit to participate in external assessment process (B.2), including publication of results. (Rec. B3) - WB, bilateral, and multilateral donors should declare that related funding will be conditional on a country’s participation in external assessment process (B.2). (Rec. B4) - IMF should include pandemic preparedness in its economic and policy assessments of individual countries, based on outcomes of the external assessment of national core capacities. (Rec. B5) - Countries should develop plans to achieve and maintain benchmark core capacities by mid-2017 (target to achieve full compliance with the benchmarks by 2020). (Rec. B6) - WHO should provide technical support to fill countries’ gaps in core capacities and achieve benchmark performance. (Rec. B7) - National governments should develop domestic resourcing plans to finance improvement and maintenance of core capacities as set out in plans (B.6). (Rec. B8) - WB should convene other multilateral donors to secure financial support for lower-middle-income and low-income countries in delivering plans (B.6). (Rec. B9) - WHO should create a CHEPR to lead the global effort toward outbreak preparedness and response. This center should be governed by an independent TGB. (Rec. C1) |
| *Harvard/LSHTM* | - Develop a global strategy to invest in, monitor, and sustain national core capacities. (Rec. 1) - Institutionalise accountability by creating an independent Accountability Commission for Disease Outbreak Prevention and Response. (Rec. 5) - Create a unified CEPR with clear responsibility, adequate capacity, and strong lines of accountability. (Rec. 3) |
| *UN Panel* | - WHO strengthens its periodic review of compliance with the IHR Core Capacity requirements. (Rec. 6) - By 2020, States Parties to the IHR, with appropriate international cooperation, are in full compliance with the IHR Core Capacity requirements. (Rec. 1) - WHODG leads urgent efforts, in partnership with the WB, Regional Development Banks, other international organizations, partners, foundations and the private sector, to mobilize financial and technical support to build IHR Core Capacities. (Rec. 17) - Regional and sub-regional organizations develop or strengthen standing capacities to monitor, prevent and respond to health crises, supported by the WHO. (Rec. 5) - UN General Assembly immediately creates a High-level Council on Global Public Health Crises to ensure the world is prepared and able to respond to public health crises. (Rec. 26) |
| *WHO Interim Assessment* | - WHO should propose a prioritized and costed plan, based on independently assessed information, to develop core capacities required under the IHR for all countries. The financing of this plan is to be done in close partnership with the WB. (Rec. 1) - WHO should establish the WHO CEPR, which will be based on the currently separate outbreak control and humanitarian areas of work. WHO CEPR will need to develop new organizational structures and procedures to achieve full preparedness and response capacity. (Rec. 11) - WHO, through the WHODG, should immediately establish an independent Board to oversee the WHO CEPR. It should guide the development of the new WHO CEPR and report on its progress to the Executive Board, Health Assembly and the UN Inter-Agency Standing Committee. The Chair of this Board should provide an annual report on global health security to the Executive Board, Health Assembly and the UN General Assembly. (Rec. 12) |
| **Goal 5: Achieve gender equality and empower all women and girls** | |
| *CGHRF* | No recommendation. |
| *Harvard/LSHTM* | No recommendation. |
| *UN Panel* | - Outbreak preparedness and response efforts should take into account and address the gender dimension. (Rec. 4) |
| *WHO Interim Assessment* | No recommendation. |
| **Goal 11.5: By 2030, significantly reduce the number of deaths and the number of people affected and substantially decrease the direct economic losses relative to global gross domestic product caused by disasters, including water-related disasters, with a focus on protecting the poor and people in vulnerable situations** | |
| *CGHRF* | - WHA should agree on new mechanisms for holding governments publicly accountable for performance under the IHR and broader GHRF, including protocols for avoiding delays in data and alerts and unnecessary restrictions on trade or travel. (Rec. C.8) - IMF should ensure capability to provide budgetary support to governments raising alerts of outbreaks. (Rec. C.10) |
| *Harvard/LSHTM* | - Strengthen incentives for early reporting of outbreaks and science-based justifications for trade and travel restrictions. (Rec. 2) |
| *UN Panel* | - WTO and WHO convene an informal joint Commission of Experts to study possible measures to strengthen coherence between the IHR and the WTO legal frameworks regarding trade restrictions imposed for public health reasons. (Rec. 24) - IHR Review Committee considers developing mechanisms to rapidly address unilateral action by states and others who are in contravention of temporary recommendations issued by the WHO as part of a PHEIC announcement. (Rec. 23) |
| *WHO Interim Assessment* | - The IHR Review Committee for Ebola should consider incentives for encouraging countries to notify public health risks to WHO. These might include innovative financing mechanisms such as insurance triggered to mitigate adverse economic effects. (Rec. 3) - IHR Review Committee for Ebola should consider disincentives to discourage countries from taking measures that interfere with traffic and trade beyond those recommended by WHO. (Rec. 4) |
| **Goal 13.1: Strengthen resilience and adaptive capacity to climate-related hazards and natural disasters in all countries** | |
| *CGHRF* | - UNSG should work with the WHO and other parts of the UN system to develop strategies for sustaining health system capabilities and infrastructure in fragile and failed states and in war zones, to the extent possible. (Rec. B.10) |
| *Harvard/LSHTM* | No recommendation. |
| *UN Panel* | - Partners sustain their Official Development Assistance to health and direct a greater percentage to strengthening health systems under an agreed-upon government-led plan. (Rec. 3) |
| *WHO Interim Assessment* | No recommendation. |

**Abbreviations used in Supplementary Table 2**

| *CEPR* | Centre for Emergency Preparedness and Response | *PPDC* | Pandemic Product Development Committee |
| --- | --- | --- | --- |
| *CHEPR* | Center for Health Emergency Preparedness and Response | *R&D* | research and development |
| *GHRF* | Global Health Risk Framework | *Rec.* | recommendation |
| *GHSA* | Global Health Security Agenda | *TGB* | Technical Governing Board |
| *IHR* | International Health Regulations | *UN* | United Nations |
| *IMF* | International Monetary Fund | *UNSG* | UN Secretary General |
| *NFP* | National Focal Point | *WB* | World Bank |
| *OCHA* | Office for the Coordination of Humanitarian Affairs | *WHA* | World Health Assembly |
| *OIE* | World Organization for Animal Health | *WHO* | World Health Organization |
| *PEF* | Pandemic Emergency Facility, | *WHODG* | WHO Director-General |
| *PHEIC* | Public Health Emergency of International Concern | *WTO* | World Trade Organization |
